# Mirvetuximab Soravtansine in the Treatment of Chemotherapy-Resistant Ovarian Cancer: A Systematic Review

**DOI:** 10.3390/ijms27135887

**Published:** 2026-06-30

**Authors:** Natalia Picheta, Julia Piekarz, Jakub Pobideł, Karolina Daniłowska, Natalia Gierulska, Krzysztof Kułak, Anna Kułak, Ewa Tomaszewska, Iwona Puzio

**Affiliations:** 1 Student’s Scientific Association—I Chair and Department of Gynecological Oncology and Gynecology, Medical University of Lublin, Staszica 16 Str., 20-081 Lublin, Poland; natalia.picheta2812@gmail.com (N.P.); jakubpod@gmail.com (J.P.); karolina02812@gmail.com (K.D.); gierulskanatalia@gmail.com (N.G.); 2 I Chair and Department of Gynecological Oncology and Gynecology, Medical University of Lublin, Staszica 16 Str., 20-081 Lublin, Poland; 3 Department of Diagnostic and Microsurgery of Glaucoma, Medical University of Lublin, 20-059 Lublin, Poland; puzioan@gmail.com; 4 Department of Animal Physiology, Faculty of Veterinary Medicine, University of Life Sciences, Akademicka 13, 20-950 Lublin, Poland; ewa.tomaszewska@up.edu.pl (E.T.); iwona.puzio@up.edu.pl (I.P.)

**Keywords:** mirvetuximab soravtansine, ADC, platinum-resistant ovarian cancer, folate receptor α, chemotherapy

## Abstract

Ovarian cancer is a major cause of gynecological cancer mortality, frequently associated with platinum-resistant recurrences. Given the limited efficacy of conventional chemotherapy in this setting, alternative targeted therapeutics are needed. Mirvetuximab soravtansine (MIRV) is an antibody–drug conjugate designed to deliver the cytotoxic maytansinoid DM4 to folate receptor alpha (FRα)-overexpressing cells. This systematic review of PubMed, ClinicalKey, and SpringerLink databases (2019–2026) evaluates five publications across three clinical trials (one phase II, two phase III) encompassing 925 patients with platinum-resistant disease. Notably, the phase III MIRASOL trial demonstrated improved survival outcomes with MIRV over standard chemotherapy, extending median overall survival (16.46 vs. 12.75 months; HR 0.67) and progression-free survival (5.62 vs. 3.98 months; HR 0.65), with an objective response rate (ORR) of 42.3% versus 15.9%. Furthermore, the single-arm phase II SORAYA trial reported an ORR of 32.4% in pretreated patients, including those with prior PARP inhibitor and bevacizumab exposure. Although the preceding FORWARD I trial missed its primary endpoint in the unselected population, its high-FRα subgroup analysis revealed a clinical benefit that influenced subsequent biomarker-driven enrollment strategies. From a safety perspective, MIRV exhibited lower rates of severe neutropenia and anemia than chemotherapy, with toxicities primarily consisting of manageable, reversible ocular events. Ultimately, MIRV serves as a therapeutic option for platinum-resistant, FRα-positive ovarian cancer, offering survival advantages; however, rigorous biomarker-based screening remains necessary to optimize therapeutic outcomes.

## 1. Introduction

Ovarian cancer is the second most common cause of death among gynecological malignancies in women. It accounts for approximately 2.1% of all cancer-related deaths, and about 313,959 new cases are diagnosed worldwide each year. This malignancy is most commonly diagnosed in women aged 55–70 years, with the peak incidence occurring between 55 and 59 years of age. A major challenge in the management of ovarian cancer is its late diagnosis; approximately 70% of cases are diagnosed at an advanced stage, such as FIGO stage III or FIGO stage IV [1]. An important aspect to consider is screening for the early detection of ovarian cancer, including ultrasonography and the tumor marker in serum cancer antigen 125 (CA-125); however, these strategies have not been shown to improve overall survival (OS) [2,3].

Various factors predispose individuals to the development of ovarian cancer, including nulliparity, endometriosis, the postmenopausal period, obesity, talc use, and tobacco exposure [4]. Genetic predisposition associated with mutations in the breast cancer type 1 susceptibility protein (*BRCA1*) gene increases the risk of ovarian cancer by approximately 40–60%, whereas breast cancer type 2 susceptibility protein (*BRCA2*) mutations increase the risk by about 11–27%. The mutations are present in approximately 18% of women diagnosed with ovarian cancer [5].

Protective factors associated with a reduced risk of ovarian cancer include pregnancy, breastfeeding, and the use of oral contraceptives [6].

At the time of diagnosis, approximately 95% of patients present with nonspecific abdominal complaints, including bloating, urinary frequency, abdominal pain, and urinary urgency. In patients with more advanced stages of the disease, ascites and the presence of an abdominal mass may also occur.

The treatment of ovarian cancer in the early stages primarily involves surgical management, including hysterectomy, bilateral salpingo-oophorectomy, omentectomy, and lymphadenectomy, followed by adjuvant chemotherapy consisting of carboplatin and paclitaxel. The 5-year survival rate after this treatment ranges from approximately 70% to 95% [7].

In more advanced stages of the disease, treatment consists of primary cytoreductive surgery followed by adjuvant chemotherapy with carboplatin and paclitaxel, or neoadjuvant chemotherapy. At a later stage of treatment, interval cytoreductive surgery may be performed, followed by additional adjuvant chemotherapy. In advanced ovarian cancer, the 5-year survival rate is approximately 10–40%. Maintenance therapy may include bevacizumab or poly (ADP-ribose) polymerase (PARP) inhibitors [8].

However, these treatment strategies do not always achieve satisfactory outcomes; therefore, there is a need to develop novel therapeutic approaches with improved efficacy that may ultimately lead to prolonged survival.

In light of the aforementioned considerations, this review highlights mirvetuximab as a promising targeted therapy. Mirvetuximab is an antifolate receptor 1 (FOLR1) monoclonal antibody. The drug is an antibody–drug conjugate (ADC), specifically mirvetuximab soravtansine (MIRV), targeting the folate receptor alpha (FRα) [9].

The drug demonstrates particular benefit in patients with high FRα expression who are resistant to platinum-based therapy. In clinical studies, it has shown a significant advantage compared with chemotherapy in terms of progression-free survival (PFS), OS and objective response rate (ORR). This systematic review formally synthesizes data from pivotal trials strictly evaluating mirvetuximab soravtansine monotherapy in platinum-resistant disease. Section 4 will also contextually address emerging data from related studies in platinum-sensitive settings and combination regimens.

## 2. Materials and Methods

### 2.1. Study Design

The systematic review was conducted in accordance with the Preferred Reporting Items for Systematic Reviews and Meta-Analyses (PRISMA) guidelines. The aim of the review was to comprehensively assess the efficacy and safety of MIRV in the treatment of chemotherapy-resistant ovarian cancer.

The analysis encompassed prospective clinical trial publications evaluating MIRV monotherapy in adult patients with platinum-resistant ovarian cancer (PROC), comprising randomized phase III trials, a single-arm phase II study, final survival follow-up reports, post hoc subgroup analyses, and patient-reported outcome (PRO) analyses derived from the parent trial programs.

### 2.2. Eligibility Criteria and PICO Framework

The study focused on assessing the efficacy and safety of MIRV. The Population, Intervention, Comparison, Outcome (PICO) model was utilized to organize and guide the literature review:

Population: The target population comprised patients with histologically confirmed epithelial ovarian cancer exhibiting platinum-resistant disease (defined as radiologically documented disease progression during or within 6 months of completing the last platinum-based therapy). Eligible cohorts were required adequate FRα expression on tumor cells (with positivity thresholds of >50% or ≥75% of viable tumor cells, depending on the specific trial protocol) and a history of 1 to 3 lines of systemic therapy. Additionally, an Eastern Cooperative Oncology Group (ECOG) performance status of 0 or 1 and at least one measurable lesion according to the Response Evaluation Criteria in Solid Tumors (RECIST) 1.1.

Intervention: Evaluation was restricted to the administration of intravenous MIRV monotherapy at the protocol-specified dose of 6 mg/kg per cycle.

Comparison: The efficacy and safety of MIRV therapy were compared with standard chemotherapy of the investigator’s choice—paclitaxel, pegylated liposomal doxorubicin (PLD), or topotecan. For single-arm studies, therapeutic efficacy was investigated without a concurrent control group.

Outcome: The synthesized data indicated that the investigational conjugate provides measurable clinical benefits in patients with FRα-overexpressing, platinum-resistant ovarian cancer compared with standard chemotherapy. The treatment significantly improved ORR and, under rigorous selection criteria for high receptor expression, prolonged both PFS and OS. Furthermore, this approach was associated with a distinct, more favorable, and manageable toxicity profile, demonstrating a significant reduction in severe adverse events, particularly bothersome hematologic toxicities.

Studies published between 2019 and 2026 were eligible for the analysis. Phase III randomized controlled trials (RCTs) and single-arm phase II studies were included if they contained data on the use of MIRV in the patient population in question. Exclusion criteria included review publications, case reports, retrospective analyses, reports of preclinical studies (both in animal and cellular models), phase I studies, and papers that did not include information on the efficacy or safety of MIRV or focused on populations other than ovarian cancer.

### 2.3. Search and Selection Process

The search terms in the databases: PubMed, ClinicalKey, and SpringerLink covered the years 2019–2026 to provide the most up-to-date data. The systematic search was conducted using precise, database-specific search strings combining terms for the intervention and target disease. The exact search queries used were as follows:

- PubMed: (“Mirvetuximab soravtansine” OR “Elahere” OR “IMGN853” OR “ADC”) AND (“Ovarian cancer” OR “Platinum-resistant ovarian cancer”)
- ClinicalKey: (“Mirvetuximab soravtansine” OR “Elahere” OR “IMGN853” OR “ADC”) AND (“Ovarian cancer” OR “folate receptor α”)
- SpringerLink: (“Mirvetuximab soravtansine” OR “Elahere” OR “IMGN853”) AND (“Chemotherapy”) AND (“Ovarian cancer”)

**Figure 1:**
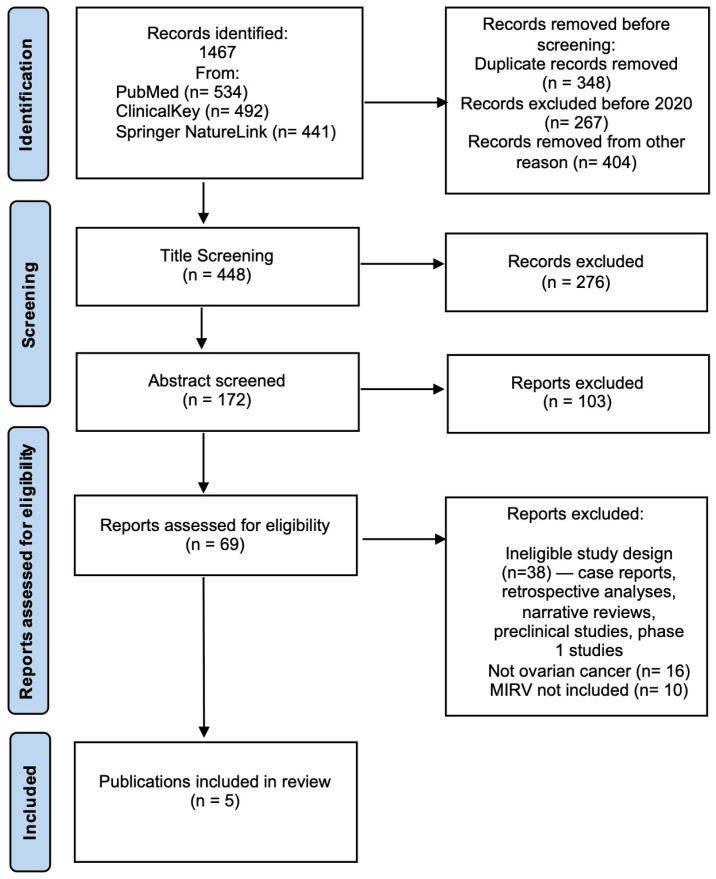
PRISMA (Preferred Reporting Items for Systematic Reviews and Meta-Analyses) flowchart for identifying, including, and excluding studies.

The final systemic search was conducted on 16 March 2026. For comprehensive coverage, a manual search of the results and an evaluation of the bibliographies of the selected articles were also conducted. Two reviewers independently screened the databases and critically appraised the selected articles. Any discrepancies were resolved through verification by other authors. A systematic database search identified 1467 records. Prior to screening, 1019 records were removed: 348 duplicates (identified and removed using Zotero v7.0, Corporation for Digital Scholarship, Vienna, VA, USA), 267 records published before 2019, and 404 records excluded by automated database filters (e.g., non-English language publications, non-journal articles). The remaining 448 records underwent title screening, resulting in the exclusion of 276 items. Out of 172 abstracts evaluated, 103 were excluded as irrelevant to the core research questions, leaving 69 reports for full-text eligibility assessment. Following a comprehensive full-text review, 64 reports were excluded (38 due to ineligible study design, 16 due to non-ovarian cancer populations, and 10 due to the absence of MIRV evaluation), yielding 5 publications describing 3 unique clinical trials that fulfilled all eligibility criteria for final synthesis. The review protocol was prospectively registered in the International Prospective Register of Systematic Reviews (PROSPERO) database under registration number [CRD420261408642]. The comprehensive data selection and identification process is illustrated in Figure 1.

### 2.4. Data Extraction

The review included one single-arm phase II study (SORAYA, reported across two publications for primary outcomes and final survival/post hoc analysis) and two phase III RCTs: FORWARD I and MIRASOL (primary publications and another that analyzes quality of life). Data extraction was performed independently by two reviewers using a standardized data extraction form. Extracted data included study characteristics (author, year, study design), patient demographics (total number of patients), intervention details (MIRV, dosing regimen), and outcome measures related to efficacy—ORR, PFS, OS, duration of response (DoR), CA-125 response and safety (assessment of the incidence of adverse events, including serious adverse events grade ≥ 3, specific ocular and hematological toxicities, and events leading to dose reduction or treatment discontinuation). Any discrepancies were resolved through discussion or consultation with other reviewers.

### 2.5. Risk of Bias Assessment of Included Studies

**Figure 2:**
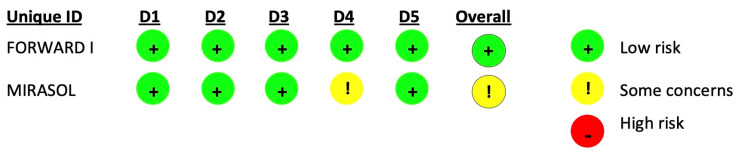
Risk of bias assessment for randomized controlled trials included in the review, evaluated using the Cochrane Risk of Bias 2.0 (RoB 2.0) tool.

The risk of bias was assessed independently by two reviewers. Discrepancies were resolved through discussion, with a third reviewer acting as adjudicator when necessary. Two randomized controlled trials (FORWARD I, MIRASOL) were assessed using the Cochrane Risk of Bias 2.0 (RoB 2.0) tool, which assesses five areas: (1) randomization process, (2) deviations from intended interventions, (3) missing outcome data, (4) outcome measurement, and (5) choice of reported outcome. Each area was rated as low risk, high risk, or with some concern. The detailed results of the risk of bias assessment for the randomized controlled trials are summarized in Figure 2.

The randomized controlled trials, both utilizing an open-label design, demonstrated distinct risk profiles upon evaluation. The FORWARD I trial was graded as having a low risk of bias across all domains, as the open-label nature was mitigated by a blinded independent central review of the primary endpoints. Conversely, the MIRASOL trial was rated with ‘some concerns’ overall, driven specifically by Domain 4 (measurement of the outcome) due to investigator-assessed and subjective patient-reported endpoints, which introduce a risk of detection bias.

For the non-randomized evidence, the single-arm phase II SORAYA trial achieved a maximum score of 16 out of 16 points on the Methodological Index for Non-Randomized Studies (MINORS) scale, indicating complete reporting according to the protocol criteria. However, because this trial lacks a concurrent control group, its non-comparative design inherently limits the validity of its efficacy and safety findings relative to standard cytotoxic regimens.

## 3. Results

### 3.1. Mirvetuximab Soravtansine

Platinum resistance in ovarian cancer is a multifactorial process, and the mechanisms leading to its development have not been fully elucidated. Therefore, novel therapeutic strategies based on targeted therapies are being explored. ADCs represent a class of agents that combine the cytotoxic activity of chemotherapy with the selective targeting of tumor-specific antigens. It consists of a monoclonal antibody directed against tumor-associated antigens and a cytotoxic payload [10].

**Figure 3:**
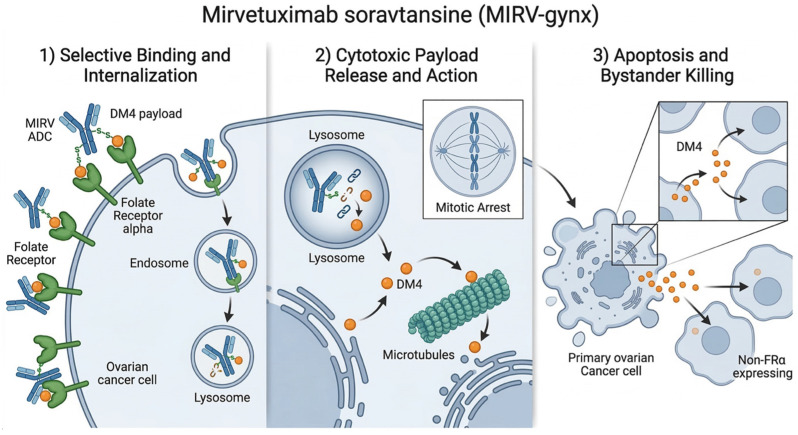
Mechanism of action of mirvetuximab soravtansine [14,15]. The conjugate selectively binds to folate receptor alpha (FRα) on the cancer cell surface and is then internalized. Inside the cell, a cytotoxic payload, DM4, is released, which inhibits microtubule dynamics, leading to cell cycle arrest and apoptosis. Furthermore, the released DM4 molecules diffuse into neighboring cells, causing their death, even if they do not express FRα [14,15].

MIRV is an ADC that contains a chimeric IgG1 monoclonal antibody binding to FRα, conjugated to the cytotoxic effector molecule maytansinoid DM4 via a charged, cleavable disulfide linker [11]. This drug is directed against the aforementioned receptor, mediating its cytotoxic effect through a tubulin-targeting payload. Following this interaction, the resulting antibody–antigen complex undergoes rapid internalization, leading to the intracellular release of DM4 [12]. DM4 induces cell cycle arrest and apoptosis through inhibition of microtubule dynamics, thereby exerting potent antimitotic activity [13]. Due to its structural properties, the drug also produces a so-called “bystander killing” effect, which involves the diffusion of active DM4 metabolites, cleaved from the MIRV construct, from antigen-positive tumor cells to neighboring cells, ultimately inducing their apoptosis [14,15]. Figure 3 shows the mechanism of action of MIRV.

The role of FRα in tumor development and survival has not been fully established. Nevertheless, its overexpression on the surface of tumor cells makes it an attractive target for ADCs, particularly when the receptor’s presence on epithelial ovarian malignant cells reaches ≥90%. Additionally, low expression levels of FRα may be associated with failure of first-line chemotherapy [16,17]. Therefore, the selection of the antigen targeted by an ADC is crucial, as effective therapy requires high expression on malignant cells and minimal expression on normal tissues.

MIRV-gynx received accelerated approval by the U.S. Food and Drug Administration (FDA) on 14 November 2022, based on the single-arm phase II SORAYA trials, for the treatment of adults with FRα-positive PROC who had undergone one to three prior lines of systemic therapy. On 22 March 2024, the FDA granted full approval based on the confirmatory phase 3 MIRASOL trial [18]. The European Medicines Agency (EMA) approved MIRV-gynx on 14 November 2024 for use in adult patients with advanced ovarian cancer whose tumor cells express FRα on their surface, in cases where the disease has become resistant to platinum-based therapy [19].

### 3.2. Research

#### 3.2.1. SORAYA

The first study is the SORAYA study—a single-arm phase II clinical trial [20]. The study enrolled 106 patients, of whom 105 constituted the efficacy population. Inclusion criteria included histologically confirmed ovarian cancer, FRα expression (requiring ≥75% viable tumor cells), 1–3 lines of prior systemic therapy, and, according to the protocol, prior bevacizumab use was absolutely necessary. 51% of patients had received 3 lines of therapy, and 48% had previously received PARP inhibitors. MIRV was administered at a dose of 6 mg/kg in cycles every 3 weeks.

The primary endpoint was ORR, which was 32.4% (95% CI, 23.6–42.2). Complete remission (CR) was achieved in 5 patients (4.8%), and partial remission (PR) was achieved in 29 patients (27.6%). Tumor shrinkage was observed in over 71% of patients.

The median DOR was 6.9 months (95% CI, 5.6–9.7). Median PFS was 4.3 months, and OS was 13.8 months. Dose reduction was required in 20% of patients. The most common adverse events were blurred vision (41% of all grades; 6% grade 3) and keratopathy (29% of all grades; 8% grade 3 and 1% grade 4). These adverse events resolved in 96% of patients after dose reduction and steroid drops. Nausea was also frequently observed (29%), but was not severe (0% grade ≥ 3) [20].

The next study is a continuation of the SORAYA study, which summarizes the final OS results and provides a post hoc analysis [21]. The final OS was 15 months (95% CI, 11.5–18.7). As many as 37% of patients survived 2 years after starting treatment. Patients who had received one or two prior lines of therapy had a median OS of an excellent 18.7 months. Patients with three prior lines of therapy had a median OS of 11.6 months [21].

#### 3.2.2. FORWARD I

This is a randomized, phase 3 clinical trial that enrolled 366 patients with histologically confirmed epithelial ovarian cancer, fallopian tube cancer, or primary peritoneal cancer [22]. Inclusion criteria included: platinum resistance, defined as disease progression within 6 months of completing platinum-based therapy, prior treatment (patients had received 1–3 lines of systemic therapy), FRα expression confirmed by immunohistochemistry (IHC) (requiring a positivity threshold of more than 50% of tumor cells), ECOG performance status of 0 or 1, and at least one measurable lesion according to RECIST 1.1. Patients were assigned to the MIRV group (*n* = 243) and the chemotherapy (CTH) group (*n* = 109) in a 2:1 ratio. MIRV was administered at a dose of 6 mg/kg intravenously every 21 days, and CTH in one of 3 doses: paclitaxel 80 mg/m^2^ (days 1, 8, 15; 4-week cycle), PLD 40 mg/m^2^ (day 1; 4-week cycle), or topotecan: 4 mg/m^2^ (days 1, 8, 15; 4-week cycle) or 1.25 mg/m^2^ (days 1–5; 3-week cycle) [22]. The primary endpoint was PFS assessed in the overall population and the subgroup with high FRα expression. No statistically significant difference was demonstrated for the overall population: median PFS was 4.1 months for MIRV and 4.4 months for CTH (HR 0.98, *p* = 0.897). In the high FRα expression population, a trend toward benefit was observed for the MIRV group with a median of 4.8 months and the CTH group with a median of 3.3 months (HR 0.69), but this did not reach statistical significance for this procedure (*p* = 0.063) [22]. Secondary outcomes are presented in Table 1.

MIRV demonstrated a more favorable and better-managed toxicity profile compared with CTH. This conjugate was associated with a significantly lower incidence of grade 3 treatment-related adverse events, which occurred in 25.1% of patients compared with 44% in the control group. This translated into fewer dose reductions (19.8% vs. 30.3%) and lower rates of treatment discontinuation due to toxicity (4.5% vs. 8.3%) [22].

The specific toxicity profile of MIRV primarily included ocular disturbances, primarily manifesting as blurred vision (42%) and keratopathy (32.5%), with the majority of these events being mild (grade 3 in 2% and 1.2% of patients, respectively). The most common systemic side effects included nausea (45.7%) and diarrhea (31.3%), which were effectively controlled with standard supportive care. A particularly significant difference was noted in hematologic toxicity, with MIRV demonstrating a significant advantage over chemotherapy in the incidence of neutropenia (6.6% vs. 39.4%) and anemia (10.7% vs. 28.4%). Furthermore, no treatment-related deaths were reported in the MIRV arm, whereas two such deaths due to sepsis occurred in the chemotherapy group [22].

#### 3.2.3. MIRASOL

The MIRASOL study is a randomized controlled trial involving 453 patients [23]. Inclusion criteria included platinum resistance—radiologically confirmed disease progression during or after the last platinum-based therapy, patients who had received 1–3 lines of systemic therapy, FRα expression defined as the presence of the receptor on ≥75% of viable tumor cells, ECOG performance status of 0 or 1, and at least one measurable lesion according to RECIST 1.1 criteria. Patients were randomized 1:1 to receive MIRV (*n* = 227) at a dose of 6 mg/kg intravenously every 3 weeks and CTH (*n* = 226), paclitaxel (80 mg/m^2^ intravenously on days 1, 8, 15, and 22 of a 4-week cycle), PLD (40 mg/m^2^ intravenously on day 1 of a 4-week cycle), or topotecan (4 mg/m^2^ intravenously on days 1, 8, and 15 of a 4-week cycle or 1.25 mg/m^2^ on days 1–5 of a 3-week cycle) [23].

The primary endpoint was PFS, with a median of 5.62 months in the MIRV arm and 3.98 months in the CTH arm (*p* < 0.001). Median OS was 16.46 months in the MIRV arm compared with 12.75 months in the CTH arm. This translated into a 33% reduction in the risk of death (HR 0.67; *p* = 0.005). The ORR was 42.3% in the MIRV arm compared with only 15.9% in the CTH arm. The odds ratio (OR) for response was 3.81 (*p* < 0.001). Of note, 12 patients (5.3%) in the MIRV arm achieved CR, whereas none in the chemotherapy arm [23].

MIRV was associated with a different and generally more favorable toxicity profile compared with conventional chemotherapy. Adverse events of grade ≥ 3 occurred in 41.7% of patients treated with MIRV compared with 54.1% in the CTH arm. Serious adverse events were reported in 23.9% of MIRV and 32.9% of CTH patients, respectively. MIRV had a significantly lower rate of permanent treatment discontinuation due to toxicity, at 9.2%, compared with 15.9% in the CTH arm. The most common adverse events of any grade in the MIRV arm were visual disturbances: blurred vision (40.8%) and keratopathy (32.1%), followed by abdominal pain (30.3%) and fatigue (30.3%). In the CTH group, bothersome hematological toxicity predominated, including primarily anemia (34.3%) and neutropenia (28.5%), as well as nausea (29%) and fatigue (25.1%) [23].

The second study is a continuation of the MIRASOL study. The primary endpoint was improvement in gastrointestinal symptoms. 21% of patients treated with MIRV (34 of 162) reported improvement, compared with 15.3% of patients (23 of 150) treated with investigator’s choice chemotherapy, but this difference was not statistically significant (OR 1.5 [95% CI 0.8–2.6]; *p* = 0.26) [24]. Additionally, in the MIRV group, mean abdominal and gastrointestinal symptom scores decreased—signifying improvement—at all time points up to week 24.

Fatigue was assessed using a standardized and validated oncology tool, the European Organization for Research and Treatment of Cancer Quality of Life Questionnaire-Core 30 (EORTC QLQ-C30). Scores on this tool range from 0 to 100, and in symptom subscales such as fatigue, higher scores correlate with more severe symptoms. A longitudinal analysis using mixed models for repeated measures (MMRM) demonstrated that the use of MIRV in patients with platinum-resistant ovarian cancer effectively prevented the progression of fatigue, which systematically worsened in the group receiving standard CHT. The difference in least squares means (LS mean) at weeks 8 or 9 of treatment was −8.4 points in favor of MIRV (*p* < 0.0001), indicating a clinically significant reduction in this symptom. This difference further widened at subsequent assessments, reaching −10.7 points at week 24 (*p* < 0.0001). Furthermore, at the strict threshold of clinical significance (defined in this study as a decrease in the fatigue subscale score of ≥22.22 points), MIRV significantly delayed the time to final deterioration in this dimension (HR 0.61; 95% CI 0.42–0.90; *p* = 0.013). The median time to deterioration was not reached (NR) in the treatment group, whereas in the control group it was 8.44 months [24].

Adequate benefits were also observed in the global quality of life (GHS-QoL) domain. For the GHS-QoL subscale, a higher score indicates better quality of life. While patients in the chemotherapy arm experienced progressive deterioration, patients treated with MIRV maintained their baseline quality of life throughout the 24-week follow-up period. The LS mean difference in GHS-QoL at weeks 8 or 9 was 7.2 points in favor of MIRV (*p* < 0.0001) and maintained the trend favoring the conjugate at subsequent time points. MIRV therapy reduced the risk of clinically significant deterioration in GHS-QoL by 32% (HR 0.68; 95% CI 0.48–0.97; *p* = 0.032), extending the median time to final deterioration from 7.16 months in the chemotherapy group to 9.07 months in the MIRV group [24]. Table 2 summarizes the most important endpoints of the studies presented in the review.

## 4. Discussion

Treatment of PROC has long been one of the greatest challenges in modern gynecologic oncology. After exhausting platinum-based treatment options and supportive therapies, patient prognosis has dramatically declined, and standard sequential CTH is characterized by disappointingly low ORR and high cumulative toxicity. With the benefits of conventional cytotoxic agents exhausted, the search for effective options to overcome multidrug resistance has become an absolute priority. The results presented in this systematic review demonstrate that MIRV provides a valuable, targeted therapeutic addition to the restrictive pharmacotherapy of this cancer. The clinical significance of these results is reflected in the current National Comprehensive Cancer Network (NCCN) guidelines, which list MIRV monotherapy as the preferred category 1 option for PROC expressing FRα (≥75% tumor cells positive) and MIRV plus bevacizumab as useful in certain circumstances for tumors with ≥25% FRα expression. In platinum-sensitive disease, MIRV is also recommended for tumors expressing FRα [25].

A methodological cross-trial comparison reveals that variations in the reported clinical efficacy of MIRV are fundamentally contingent upon patient selection criteria and biomarker expression thresholds. In the phase III FORWARD I trial, which defined folate receptor alpha (FRα) positivity as >50% of tumor cells, the conjugate failed to achieve a statistically superior progression-free survival (PFS) over standard chemotherapy within the unselected overall population. Conversely, the subsequent phase III MIRASOL trial restricted enrollment to a high-expression cohort (≥75%), a strategic refinement that yielded a statistically significant prolongation of both median PFS and OS relative to the control group. Distinct from these randomized designs, the single-arm phase II SORAYA trial reported an objective response rate (ORR) of 32.4% and a median PFS of 4.3 months. However, interpretation of the SORAYA data must account for the lack of a concurrent control arm and the protocol-mandated requirement for prior bevacizumab exposure, which establishes a distinct clinical baseline compared with the populations evaluated in FORWARD I and MIRASOL. Consequently, these findings underscore that the clinical efficacy of MIRV monotherapy is strictly confined to high-expressors and cannot be automatically extrapolated to patients with low or moderate FRα expression.

From a clinical oncology perspective, these findings provide a compelling argument for the adoption of MIRV. While extending OS remains the ultimate objective, achieving this survival benefit without exacerbating patient-reported symptom burden underscores the therapeutic value of this novel conjugate in preserving overall well-being.

Evolving clinical practices are also reflected in the pretreatment profiles across these trials, with the MIRASOL cohort containing a substantially higher proportion of patients previously exposed to PARP inhibitors and bevacizumab than FORWARD I. Importantly, clinical data confirmed that prior exposure to either therapeutic class did not compromise the efficacy of MIRV, reinforcing its utility as a robust option for refractory disease.

This durability was further corroborated by subgroup analyses from the SORAYA trial, where median overall survival remained consistent regardless of prior PARP inhibitor status. These concordant signals indicate that preceding targeted therapies do not induce cross-resistance or compromise the antimitotic efficacy of the conjugate.

Given the lack of effective alternatives and the poor prognosis for patients with platinum-resistant ovarian cancer, MIRV-based therapy represents a significant clinical development. As the authors of the SORAYA study conclude, this is the first step towards establishing a biomarker-guided therapeutic option as a new standard of care in this highly selected, refractory patient population.

The median OS for patients in the SORAYA study was higher in those who had received one or two prior lines of treatment compared with those who had received three. This reflects a well-established principle in platinum-resistant ovarian cancer: the number of prior lines of treatment is a key prognostic factor, and treatment outcomes progressively worsen as patients undergo increasingly intensive initial treatment [26]. With each subsequent line of therapy, ovarian cancer develops additional resistance mechanisms beyond platinum resistance. Tumors exposed to multiple chemotherapy regimens accumulate genetic and epigenetic changes that cause cross-resistance to different drug classes [27].

The NCCN guidelines clearly state that “patients who fail to respond to treatment and progress on two subsequent treatment regimens without evidence of clinical benefit are less likely to benefit from additional therapy” [25]. Furthermore, patients undergoing intensive pretreatment often have poorer performance status, cumulative organ dysfunction and pre-existing toxicities from prior treatment regimens that limit their ability to tolerate and respond to subsequent therapies [25]. In patients requiring multiple lines of therapy, the disease typically progresses more biologically aggressively, with shorter intervals between relapses. Approximately 60–75% of patients with relapsed disease eventually develop platinum resistance, and with each remission and relapse cycle, the platinum-free intervals shorten [28]. The fact that MIRV demonstrated efficacy even in the subgroup with three prior lines of treatment is clinically significant, as this group has very limited effective treatment options.

To fully appreciate the expanding therapeutic landscape of MIRV, it is essential to explore data extending beyond the strict boundaries of our formal systematic review. The subsequent trials—including the PICCOLO, FORWARD II, and IMGN853-0420 studies, as well as immunotherapy combinations—did not meet our formal inclusion criteria due to differing patient populations (e.g., platinum-sensitive disease) or multi-drug regimens. However, they are discussed here purely as contextual evidence to illustrate the broader clinical potential and future directions of this conjugate.

Beyond platinum-resistant indications, the PICCOLO trial highlighted MIRV’s efficacy in platinum-sensitive ovarian cancer (PSOC) with high FRα expression, demonstrating a robust ORR of 51.9% and a median PFS of 6.93 months [29]. Crucially, the conjugate maintained high antitumor activity (ORR 45.8%) even in the challenging subgroup of patients whose disease progressed within 30 days of PARP inhibitor therapy [29]. These results demonstrate that MIRV’s utility can extend into PSOC, offering an effective alternative that overcomes PARP inhibitor resistance while avoiding the cumulative toxicity associated with repeated platinum compounds [29].

Conversely, exploring combination regimens with immunotherapy has not yielded clinical synergy. An early phase Ib trial evaluating MIRV in combination with the anti-PD-1 antibody pembrolizumab in heavily pretreated, platinum-resistant patients reported an ORR of 31% and a median PFS of 4.2 months [30]. These efficacy parameters did not exceed those achieved with MIRV monotherapy alone, indicating that the clinical benefit was primarily driven by the conjugate itself. Furthermore, the addition of pembrolizumab significantly increased toxicity, generating a distinct safety signal with pneumonia developing in 25% of the study participants. These data suggest that adding checkpoint inhibition is insufficient to safely overcome immunotherapy resistance in this population.

A more promising approach to combination therapy with MIRV is its use with bevacizumab. The rationale for this combination is that bevacizumab, by normalizing vasculature, may improve intratumoral delivery of the conjugate [31]. Early phase 1b studies have shown promising efficacy of the MIRV + bevacizumab doublet in the treatment of PROC [32]. Importantly, this activity was observed regardless of FRα expression level or prior bevacizumab exposure [33].

Clinical synergy is supported by the platinum-independent FORWARD II cohort and a carboplatin-based triplet regimen in PSOC, with real-world data confirming improved progression-free survival even in lower FRα-expressing tumors [31,34]. In contrast to the pembrolizumab outcomes, these findings establish bevacizumab as the preferred combination partner for MIRV, leading to its inclusion in the NCCN guidelines and its ongoing evaluation as maintenance therapy in the phase III GLORIOSA trial [35].

Expanding upon these platinum-sensitive maintenance paradigms, the phase II IMGN853-0420 trial, presented at the SGO 2026 annual meeting, evaluated MIRV combined with carboplatin followed by MIRV monotherapy in patients with FRα-positive (≥25%) PSOC. This regimen demonstrated robust efficacy, yielding a confirmed ORR of 62.4% overall and 62.7% in the FRα ≥ 50% cohort, alongside a median DoR of 11.2 months. Crucially, clinical activity was preserved even in the challenging subgroup of patients with prior PARP inhibitor exposure, who maintained an ORR of 63.9% [36].

Despite these promising efficacy outcomes, a major limitation across the evaluated literature is the low ethnic diversity, with results predominantly derived from Caucasian populations. Given that pharmacokinetics, comorbidities, and FRα expression patterns may theoretically vary by ethnicity, the underrepresentation of minority groups highlights a notable gap in the current evidence base. Consequently, future clinical analyses and postmarketing surveillance must prioritize more inclusive demographic representation.

Ocular toxicities represent the most frequent adverse events reported in patients receiving MIRV, with 90% of grade 2 or higher blurred vision cases and 93% of grade 2 or higher keratopathy events resolving to grade 0–1 [37]. Management of these complications requires a multidisciplinary approach. A baseline ophthalmological examination, including visual acuity and slit-lamp evaluation, is mandatory. Subsequently, follow-up assessments must be repeated every other cycle for the first eight cycles [38]. The median time to onset of the most common adverse events, blurred vision and keratopathy, was 5.9 weeks and 6.7 weeks, respectively. Per protocol, dose modification was not required for ocular events that resolved to grade 1 or less within 14 days [39].

### Management and Prevention of Common Adverse Events

Mitigating ocular side effects requires a proactive approach, beginning with a baseline ophthalmologic examination before treatment, within 14 days of the first dose, and 30 days following the final administration. Non-pharmacologic preventive measures include strict avoidance of contact lenses, use of cooling eye patches during drug infusions, and daily eye hygiene with warm compresses [40]. Medication-based prophylaxis requires continuous daily use of preservative-free, lubricating eye drops throughout treatment and for 30 days after the last dose. While initial clinical trial protocols included prophylactic topical corticosteroids (e.g., one drop of 0.1% dexamethasone applied from the day before the start of the cycle until day 3) and vasoconstrictors (three drops of 0.2% brimonidine tartrate before the infusion), current Summary of Product Characteristics (SmPC) guidelines strictly limit primary pharmacologic prevention to lubricants. Preventive application of these corticosteroids is no longer recommended to avoid long-term steroid-induced complications [19].

If ocular toxicity occurs, patients must be referred to an ophthalmologist immediately, ideally within 72 h. Treatment modifications are strictly based on the severity of the adverse event. For grade 1 toxicity, MIRV treatment can be safely continued at the current dose with close monitoring of symptoms [40]. For grade 2 events, management should be withheld until symptoms resolve to baseline or grade 1. If these manifestations clear within 14 days, MIRV treatment can be resumed at the same dosage; however, if resolution takes within 14 to 28 days, therapy should be restarted at a reduced dose. The initial occurrence of a grade 3 event requires immediate intervention interruption until recovery to grade 1 or baseline (maximum 28 days), followed by a mandatory dose reduction upon resumption of treatment. Any recurrent grade 3 event or the development of grade 4 toxicity (such as corneal perforation) requires permanent discontinuation of MIRV [40,41].

Secondary prophylaxis with topical ocular corticosteroids is strictly indicated only after the patient has experienced a grade 2 or higher corneal adverse reaction, such as keratopathy, confirmed by slit-lamp examination. During all subsequent cycles of MIRV therapy, patients must be instructed to use steroid-containing eye drops, starting on the day of the infusion and continuing for the next 7 days [40]. Although the official prescribing information does not specify the exact drug, clinical trial protocols have typically used one drop of 0.1% dexamethasone in each eye or alternative formulations such as 1% prednisolone or 0.05% difluprednate. To ensure optimal absorption and avoid washout, patients must wait at least 15 min after corticosteroid administration before applying routinely used lubricating eye drops. Because topical steroids carry an independent ocular risk, patients undergoing this secondary measure should have their intraocular pressure regularly monitored and undergo comprehensive slit-lamp examinations throughout the treatment period, every other cycle or more frequently [41].

## 5. Limitations

A primary limitation of this systematic review is its reliance on a highly constrained dataset, encompassing only five publications derived from three unique clinical trials. Because these data originate almost exclusively from strictly controlled, registration-directed settings, caution must be exercised when formulating broader clinical conclusions. These pivotal trials reflect highly selected patient cohorts, and their outcomes may not fully capture the complexities or long-term safety and efficacy profiles encountered in unselected, real-world oncology practices.

It should be noted that the studies enrolled patients with an ECOG score of 0 or 1, which can be problematic in clinical practice. Due to the advanced stage at which ovarian cancer typically presents and the associated symptom burden, many patients may have a poorer performance status in real-world settings than those included in clinical trials.

The open-label design of the pivotal phase III trials (FORWARD I, MIRASOL) represents another challenge, as the lack of blinding could introduce investigator bias during PFS assessments and potentially influence subjective patient-reported quality of life questionnaires. Consequently, the MIRASOL trial was assigned a rating of ‘Some concerns’ in the overall risk of bias evaluation (Domain 4). However, this framework is considered typical for phase III oncology trials in this setting, and the strict utilization of objective RECIST 1.1 criteria effectively minimized subjective interpretation, ensuring the validity of the demonstrated clinical benefit.

Furthermore, this review incorporates studies with diverse designs—ranging from single-arm trials to phase II/III RCTs—constraining the capacity to conduct direct meta-analyses, though such heterogeneity was unavoidable given the scarcity of available MIRV data. Moreover, the relatively small sample sizes in these publications reduce the statistical power for subgroup analyses. Additionally, FRα presence varies significantly by histologic subtype; while 70.9% of high-grade serious carcinomas are positive for the receptor, its prevalence drops sharply in endometrioid (21.1%) and clear cell carcinomas (29.3%) [41]. This limits the applicability of MIRV to non-serous histologic tumors.

Finally, the limited racial and ethnic diversity within the pivotal trial cohorts restricts the broader generalizability of these findings to global populations. The MIRASOL study population (66% white, 12% Asian, 3% black) underscores that external validity may be constrained due to insufficient demographic representation.

## 6. Conclusions

MIRV serves as an important targeted therapeutic option for patients with platinum-resistant, FRα-positive epithelial ovarian cancer.

Accumulated clinical evidence demonstrates its advantages over standard single-agent chemotherapy in terms of prolonged OS, PFS, and ORR, while maintaining a higher quality of life for patients and reducing severe hematological toxicity. However, rigorous patient selection based on precise biomarker assessment is essential to maximizing these benefits. Provided that stringent biomarker selection is enforced, MIRV demonstrates the capacity to overcome acquired multidrug resistance—including following progression on PARP inhibitors and bevacizumab—offering a viable therapeutic option for a highly specified, refractory patient population. Furthermore, the activity of combination regimens incorporating angiogenesis inhibition and the efficacy demonstrated in the platinum-sensitive setting indicate noteworthy avenues for future clinical evaluation that may expand existing maintenance treatment strategies.

## Figures and Tables

**Table 1:** Summary of secondary outcomes. The first value refers to MIRV and the second to CTH [22].

Endpoints	General Population	Population with High FRα Expression
ORR	22% and 12% (*p* = 0.015)	24% and 10% (*p* = 0.014)
CA-125	51% and 27% (*p* < 0.001)	53% and 25% (*p* = 0.001)
OS median	16.4 and 14 months (HR 0.815; *p* = 0.248)	NR and 11.8 months (HR 0.618; *p* = 0.033)
PFS2 median	10 and 8.4 months (HR 0.639; *p* < 0.001)	10.1 and 8.4 months (HR 0.557; *p* < 0.001)

Objective response rate (ORR), cancer antigen 125 (CA-125) response, overall survival (OS), second progression-free survival (PFS2), hazard ratio (HR). Not reached (NR).

**Table 2:** Table summarizing the studies presented in the review [20,21,22,23,24].

Author	Year	Type of Research	Population	Intervention	Control Group	Results
Moore KN et al. [22]	2021	RCT, phase III	352	MIRV 6 mg/kg intravenously every 21 days	Paclitaxel 80 mg/m^2^, PLD 40 mg/m^2^ or topotecan: 4 mg/m^2^ or 1.25 mg/m^2^	Median PFS: For the overall population: MIRV 4.1 months CTH 4,4 months (HR 0.98, *p* = 0.897) For the FRα-positive population: MIRV—median of 4.8 months CTH—median of 3.3 months (HR 0.69, *p* = 0.063)
Moore KN et al. [23]	2023	RCT, phase III	453	MIRV 6 mg/kg intravenously every 3 weeks	Paclitaxel 80 mg/m^2^, PLD 40 mg/m^2^ or topotecan 4 mg/m^2^ or 1.25 mg/m^2^	PFS: MIRV: 5.62 months CTH: 3.98 months (*p* < 0.001) Median OS: MIRV: 16.46 months CTH: 12.75 months 33% reduction in the risk of death (HR 0.67; *p* = 0.005). ORR: MIRV: 42.3% CTH: 15.9% OR for response was 3.81 (*p* < 0.001). CR: MIRV: 5.3% CTH: none.
Matulonis UA et al. [20]	2023	Single-arm phase II study	105	MIRV 6 mg/kg intravenously every 3 weeks	-	ORR: 32.4% (95% CI, 23.6–42.2) CR: 5 patients (4.8%) PR: 29 patients (27.6%) DOR: 6.9 months (95% CI, 5.6–9.7) PFS: 4.3 months OS: 13.8 months
Coleman RL. et al. [21]	2024	Single-arm phase II study	105	MIRV 6 mg/kg intravenously every 3 weeks	-	Median OS: 15 months (95% CI, 11.5–18.7) In patients with 1 or 2 lines of prior treatment: 18.7 months (95% CI, 13.8–not estimable (NE)) In patients with 3 lines of prior treatment: 11.6 months (95% CI, 7.1–16.7)
Van Gorp T. et al. [24]	2025	RCT, phase III	453	MIRV 6 mg/kg intravenously every 3 weeks	Paclitaxel 80 mg/m^2^, PLD 40 mg/m^2^ or topotecan 4 mg/m^2^ or 1.25 mg/m^2^	Improvement in gastrointestinal symptoms: MIRV: 21% reported improvement CTH: 15.3% (OR 1.5 [95% CI 0.8–2.6]; *p* = 0.26) EORTC QLQ-C30: MIRV: LS mean difference at 8/9 weeks of −8.4 (*p* < 0.0001). Delay in time to clinical symptom worsening (HR 0.61; *p* = 0.013). GHS-QoL: LS mean difference at 8/9 weeks of 7.2 points in favor of MIRV (*p* < 0.0001). Prolongation of median time to final worsening: MIRV: 9.07 months CTH: 7.16 months

## Data Availability

No new data were created or analyzed in this study. The original data presented in the study are openly available in references [20,21,22,23,24].

## Abbreviations

The following abbreviations are used in this manuscript:

ADC	Antibody–drug conjugate
BRCA 1	Breast cancer type 1 susceptibility protein
BRCA 2	Breast cancer type 2 susceptibility protein
CA-125	Cancer antigen 125
CR	Complete remission
CTH	Chemotherapy
DOR	Duration of response
ECOG	Eastern Cooperative Oncology Group
EMA	European Medicines Agency
EORTC QLQ-C30	European Organization for Research and Treatment of Cancer Quality of Life Questionnaire-Core 30
FDA	U.S. Food and Drug Administration
FOLR1	Antifolate receptor 1
FRα	Folate receptor alpha
GHS-QoL	Global quality of life
ICD	Immunogenic cell death
IHC	Immunochromatochemistry
MINORS	Methodological Index for Non-Randomized Studies
MIRV	Mirvetuximab soravtansine
MMRM	Mixed models for repeated measures
NCCN	National Comprehensive Cancer Network
NE	Not estimable
NR	Not reached
ORR	Objective response rate
OS	Overall survival
OSF	Open Science Framework
PARP	Poly(ADP-ribose) polymerase
PD-1	Programmed death-1
PFS	Progression-free survival
PICO	Population, Intervention, Comparison, Outcome
PLD	Pegylated liposomal doxorubicin
PR	Partial remission
PRISMA	Preferred Reporting Items for Systematic Reviews and Meta-Analyses
PROC	Platinum-resistant ovarian cancer
PSOC	Platinum-sensitive ovarian cancer
RCT/RCTs	Randomized controlled trials
RECIST	Response Evaluation Criteria in Solid Tumors
RoB 2.0	Risk of Bias 2.0
SmPC	Summary of Product Characteristics
